# Case Report: Short-Term Response to First-Line Crizotinib Monotherapy in a Metastatic Lung Adenocarcinoma Patient Harboring a Novel *TPR-ROS1* Fusion

**DOI:** 10.3389/fonc.2022.862008

**Published:** 2022-04-28

**Authors:** Shuli Wei, Mangsha Hu, Yan Yang, Xiaojie Huang, Baizhou Li, Liren Ding, Pingli Wang

**Affiliations:** ^1^Department of Respiratory and Critical Care Medicine, The Second Affiliated Hospital of Zhejiang University School of Medicine, Hangzhou, China; ^2^Department of Pathology, The Second Affiliated Hospital of Zhejiang University School of Medicine, Hangzhou, China

**Keywords:** non-small cell lung cancer (NSCLC), ROS1, translocated promoter region (TPR), crizotinib, ceritinib

## Abstract

*ROS1*-rearranged patients account for 1-2% of non-small cell lung cancer (NSCLC) cases. Approximately 10 fusion partners have been discovered, while clinical practice is actively generating knowledge of new ones and their therapeutic responses. Herein, we report a patient with stage IV NSCLC that harbored a novel *TPR-ROS1* fusion, which demonstrated a rapid but short partial response to first line crizotinib and primary resistance to subsequent ceritinib. Computed tomography detected a pulmonary nodule in a 53-year-old woman who presented with persistent cough. Histopathologic and molecular examination of the tissue biopsy indicated a poorly differentiated adenocarcinoma staining negative for PD-L1 but harbored a novel translocated promoter region (*TPR*)-*ROS1* (T4:R35) gene fusion. Frontline crizotinib monotherapy elicited a rapid partial response after 1 month, although the disease progressed another 2 months later. After another 3 months of continued crizotinib treatment, the patient manifested newly emerged and enlarged lung and brain lesions. Genomic profiling still identified *TPR-ROS1* as the only aberration, while a lymph node biopsy indicated PD-L1 immunopositivity. The patient was then treated with ceritinib and progressed within 1 month. She was started on chemotherapy with pemetrexed plus carboplatin and has achieved rapid partial response as of the latest follow-up. In summary, we provided clinical evidence of a novel *TPR-ROS1* fusion and its roles as an oncogenic driver in metastatic NSCLC. To the best of our knowledge, ours is the first case to report this fusion in NSCLC. This case was characterized by a rapid yet short-term response to first line crizotinib and primary resistance to subsequent ceritinib, while no known genetic resistance mechanism was identified and other mechanisms including histologic transformation were unlikely. Future research is needed to unveil the resistance mechanism and formulate effective treatment strategies.

## Introduction

Chromosomal rearrangements leading to fusion genes that encode a chimeric protein with aberrantly elevated ROS1 kinase activity represent an established oncogenic driver in non-small cell lung cancer (NSCLC). *ROS1*-positive patients account for 1-2% of NSCLC cases (1). Multiple fusion partners have been reported for *ROS1* rearrangement, the most common of which being *CD74*, followed by *SDC4*, *EZR*, and *SLC34A2* (2). Due to structural similarity, several tyrosine kinase inhibitors (TKIs) targeting anaplastic lymphoma kinase (ALK) or neurotrophin receptor tyrosine kinase (NTRK), such as crizotinib and entrectinib, have shown remarkable clinical efficacy and are currently recommended as first- or second-line therapy for *ROS1*-positive NSCLC (3). In a phase II clinical trial of 127 East Asian patients treated with crizotinib, median progression-free survival (PFS) was 10.2 and 18.8 months in patients with and without baseline central nervous system (CNS) metastasis, respectively (4). Studies of patients after progression on these TKIs have shed light on a handful of resistance mechanisms. For crizotinib, Gainor et al. found *ROS1* resistance mutations in 53% specimens from 16 patients (5), and McCoach et al. proposed *KIT* and β-catenin mutations and HER2-mediated signaling as off-targeted mechanisms (6). Meanwhile, new fusion partners and therapeutic properties are actively discovered in the clinic, such as a recent report of a *NPM1-ROS1* fusion (7). Herein, we report a patient with stage IV NSCLC that harbored a novel *TPR-ROS1* fusion and achieved rapid but short partial response to first line crizotinib monotherapy.

## Case Presentation

**Figure 1:**
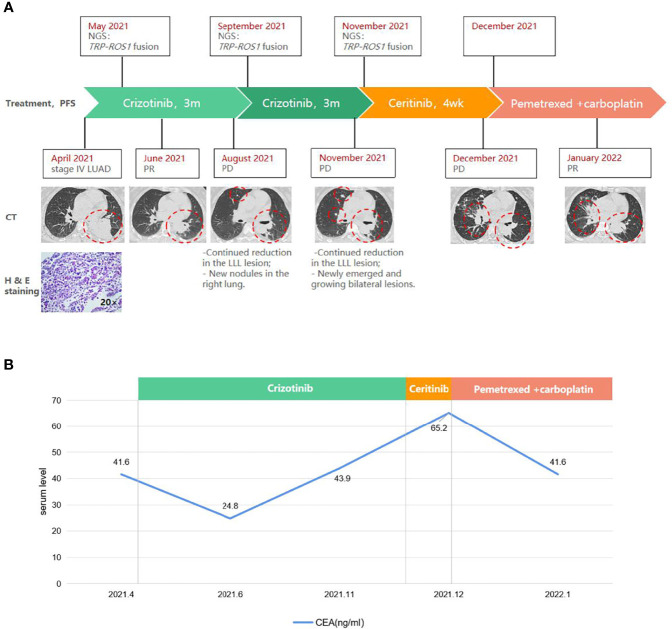
A schematic diagram of the course of management highlighting **(A)** radiographic, histopathologic, and molecular findings, and **(B)** carcinoembryonic antigen (CEA) levels at key time points. Red circles indicate the target lesion. CT, computed tomography. H & E, hematoxylin and eosin. LLL, left upper lobe. LN, lymph node. LUAD, lung adenocarcinoma. Met, metastasis. PD, progressive disease. PR, partial response. *TPR*, translocated promoter region.

**Figure 2:**
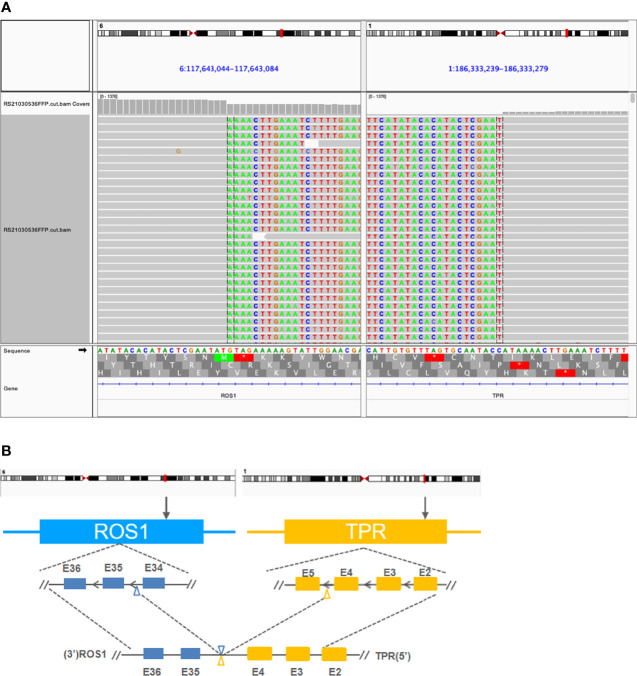
Detection of a novel *TPR-ROS1* (T4:R35) gene rearrangement using next-generation sequencing. **(A)** Identification of a *TPR-ROS1* gene fusion. **(B)** Structural illustration of the resultant putative chimeric protein. *TPR*, translocated promoter region.

A 53-year-old woman presented with persistent cough in April 2021. Past medical history was not remarkable, although the patient’s mother had lung cancer. Chest computed tomography (CT) scans detected a left lower lobe (LLL) mass and enlarged mediastinal and hilar lymph node (LN). Carcinoembryonic antigen (CEA) level was 41.6 ng/ml. A neoplasm in the left lower trachea was found on bronchoscopy, and biopsy of the neoplasm revealed a poorly differentiated adenocarcinoma (**Figure 1A**). On immunohistochemistry, the tumor stained positively for TTF1 (+++), Napsin A (+), CK7 (++), E-cadherin (++), Ki67 (50%), and negatively for P40, CD68, and PD-L1. Cancer cells were also found in biopsies of the right paratracheal, subcarinal, and mediastinal LNs. Additionally, brain magnetic resonance imaging (MRI) and enhanced CT showed a left cerebral frontal lobe mass and lesions in the T5 and L1 vertebrae. The patient was diagnosed with stage IV NSCLC (T2N3M1c). Next-generation sequencing analysis of tumor tissue and blood samples with a 168-gene panel (Burning Rock, Guangzhou, China) are as previously described (8, 9). A novel translocated promoter region (TPR)-ROS1 (T4:R35) gene fusion was detected from both samples (**Figure 2**).

Frontline treatment with crizotinib (250 mg bid) started in May and after 1 month elicited a rapid response consistent with partial response (PR) per RECIST v1.1 guidelines, manifested as a 46% reduction (59×53 mm to 31×20 mm) of the LLL mass (**Figure 1A**). CEA level also lowered to 24.8 ng/ml (**Figure 1B**). Follow-up CT in August found newly emerged right lung nodules despite continued reduction of the target lesion (25×14 mm; **Figure 1A**). Molecular testing with blood revealed similar results as baseline, with TPR-ROS1 fusion as the only alteration. As the patient was asymptomatic, crizotinib was continued. Follow-up in November showed continued reduction of the original LLL lesion (27×13 mm) but enlargement of other bilateral lung lesions and the right supraclavicular LN on CT and enlarged brain lesions on MRI, which were consistent with progressive disease (**Figure 1A**). CEA level also rose to 43.9 ng/ml. A biopsy of the right supraclavicular LN revealed poorly differentiated adenocarcinoma with immunoreactivity to PD-L1 (combined positive score 60%+). Genomic profiling of this biopsy again identified *TPR-ROS1* as the only aberration. The patient was subsequently started on ceritinib (450 mg qd) but did not appear to respond, as follow-up CT one month later indicated growing and new bilateral lung lesions and enlarged supraclavicular LN and brain metastasis, accompanied by continued rise in CEA level (65.2 ng/ml; **Figure 1**). She is now receiving a combination of pemetrexed and carboplatin and has achieved PR (sum of target lesions 46.5 mm to 31.0 mm). There was also a minor drop in CEA level (**Figure 1B**).

## Discussion

Approximately 10 genes have been reported as upstream fusion partners with *ROS1* in NSCLC (2, 7). In this case report, we provided clinical evidence of a new one. Moreover, evidence supported this novel *TPR-ROS1* (T4:R35) fusion as an oncogenic driver. The putative gene product retained the intact ROS1 kinase domain (**Figure 2**). Also, this rearrangement was identified with targeted sequencing using a moderately sizable panel (**Supplementary Table S1**) as the sole genomic abnormality prior to any treatment and after progression on crizotinib and on ceritinib. *TPR-ROS1* fusion was recently identified in a patient with lipofibromatosis, a rare pediatric soft tissue tumor (10). More interestingly, *TPR* is also known to partner with other driver genes in NSCLC. *MET* was originally identified as a proto-oncogene after molecular cloning of *TPR-MET* from chemically transformed osteosarcoma cell lines (11). Choi et al. identified *TPR-ALK* in a 60-year-old male Korean smoker who underwent lobectomy. He then received adjuvant chemotherapy with vinorelbine and cisplatin and displayed no evidence of disease as of an 18-month follow-up (12). *TPR-NTRK1* fusions have also been reported in thyroid carcinoma (13), pancreatic cancer (14), and spindle cell neoplasm, a mesenchymal tumor (15). Along with reports of *TPR-RAF* and *TPR-FGFR1*, these findings highlight *TPR* as a promiscuous fusion partner with pivotal kinases in cancer biology, although there is a dearth of knowledge regarding how patients carrying these rearrangements responded to TKI treatment.

Another noteworthy aspect of our case is the rapid progressions on crizotinib and on ceritinib. After initial PR at one month since treatment initiation, the disease progressed another two months later, leading to a PFS of 3 months. In addition to reduced inhibitory potency compared with next-generation ROS1 inhibitors, progression on crizotinib results from acquisition of resistance mechanism and/or development of CNS disease (1), which are not uncommon in *ROS1*-positive NSCLC. Patil et al. reported that CNS was the first and sole site of progression in 47% (9/19) of *ROS1*-rearranged stage IV patients (16). On the other hand, the disease did not respond to ceritinib, which unlike crizotinib, demonstrates remarkable CNS penetration. While it was possible that the patient experienced progression on first line crizotinib because of limited intracranial activity, our findings suggested existence of unidentified mechanisms driving resistance to ceritinib. Liu et al. recently reported upregulation of PD-L1 in bronchial epithelial cells after expression of *ROS1* fusion protein, which was also modulated by MEK-ERK signaling in crizotinib-resistant *ROS1*-rearragned NSCLC cells (17). It is therefore interesting to study the role of MEK-ERK signaling in mediating therapeutic resistance in our case and the efficacy of MEK inhibitors, which is the goal of our ongoing cell model experiments.

In summary, we provided clinical evidence of a novel *TPR-ROS1* fusion and its role as an oncogenic driver in metastatic NSCLC. This case was characterized by a rapid yet short-term response to first line crizotinib and primary resistance to subsequent ceritinib, while no known genetic resistance mechanism was identified and histologic transformation was unlikely. We found upregulated PD-L1 in a metastatic lesion compared with the primary after progression on crizotinib, suggesting PD-L1 increases as a potential resistance mechanism, although the possibility of inter-tumoral heterogeneity in PD-L1 expression is there. Possible mechanisms include MEK-ERK signaling, which has been reported *in vitro*, and warrant further mechanistic and clinical investigations.

## Data Availability Statement

The raw data supporting the conclusions of this article will be made available by the authors, without undue reservation.

## Ethics Statement

The studies involving human participants were reviewed and approved by Ethics Committee for Human Research of The Second Affiliated Hospital of Zhejiang University School of Medicine. The patients/participants provided their written informed consent to participate in this study. Written informed consent was obtained from the patient for publication of this case report and any accompanying images in an anonymized manner.

## Author Contributions

PW conceived of and designed the study. SW, MH, YY, and XH collected and analyzed the data. SW and PW wrote the manuscript. BL provided pathological analysis. LD provided valuable intellectual input to the manuscript and provided administrative supervision. All authors approved the final version of the manuscript and are accountable for all aspects of the work.

## Funding

This study was supported by the Key Science Project of Zhejiang Province (No. WKJ-ZJ-2122).

## Conflict of Interest

The authors declare that the research was conducted in the absence of any commercial or financial relationships that could be construed as a potential conflict of interest.

## Publisher’s Note

All claims expressed in this article are solely those of the authors and do not necessarily represent those of their affiliated organizations, or those of the publisher, the editors and the reviewers. Any product that may be evaluated in this article, or claim that may be made by its manufacturer, is not guaranteed or endorsed by the publisher.
