# Supplementary material for: Case Report: Short-Term Response to First-Line Crizotinib Monotherapy in a Metastatic Lung Adenocarcinoma Patient Harboring a Novel TPR-ROS1 Fusion
Source: Front Oncol. 2022 Apr 28;12:862008. doi: 10.3389/fonc.2022.862008 (PMC9096128; doi:10.3389/fonc.2022.862008)
Supplement: Supplementary file 1 [file Table_1.docx]

Table S1. The 168 cancer-related genes included in the targeted gene panel used in this study.

| *AKT1* | *ALK* | *APC* | *AR* | *ARID1A* | *ATM* | *ATR* | *B2M* |
| --- | --- | --- | --- | --- | --- | --- | --- |
| *BARD1* | *BCL2L11* | *BCOR* | *BLM* | *BRAF* | *BRCA1* | *BRCA2* | *BRINP3* |
| *BRIP1* | *CARD11* | *CASP8* | *CBL* | *CCND1* | *CCNE1* | *CD274* | *CD74* |
| *CDH18* | *CDK4* | *CDK6* | *CDKN1A* | *CDKN1B* | *CDKN2A* | *CHEK1* | *CHEK2* |
| *CREBBP* | *CSMD3* | *CTNNB1* | *CYP2A6* | *DIS3* | *DNMT3A* | *DPYD* | *EGFR* |
| *EMSY* | *EP300* | *EPHA3* | *EPHA5* | *EPHA7* | *EPHB1* | *ERBB2* | *ERBB3* |
| *ERBB4* | *ESR1* | *FANCA* | *FANCI* | *FAT3* | *FBXW7* | *FGF19* | *FGF3* |
| *FGF4* | *FGFR1* | *FGFR2* | *FGFR3* | *FLT1* | *FLT3* | *FLT4* | *GATA2* |
| *GATA3* | *GRIN2A* | *H3F3C* | *HGF* | *HIST1H1C* | *HIST1H3B* | *HIST1H3G* | *HRAS* |
| *IDH1* | *IDH2* | *IGF2* | *IKZF1* | *IL7R* | *INHBA* | *JAK1* | *JAK2* |
| *KDM5A* | *KDM6A* | *KDR* | *IGF1* | *IGF1R* | *KEAP1* | *KIT* | *KMT2D* |
| *KRAS* | *LRP1B* | *MAP2K1* | *MAP3K13* | *MAX* | *MCL1* | *MEN1* | *MET* |
| *MRE11* | *MSH2* | *MSH6* | *MTOR* | *MUTYH* | *MYC* | *MYCN* | *NAV3* |
| *NBN* | *NF1* | *NOTCH1* | *NRAS* | *NRG1* | *NTRK1* | *NTRK2* | *NTRK3* |
| *PAK5* | *PALB2* | *PARP1* | *PDGFRA* | *PDGFRB* | *PIK3C2G* | *PIK3C3* | *PIK3CA* |
| *PIK3CG* | *PIK3R1* | *PMS2* | *POLD1* | *POLE* | *POM121L12* | *PPP2R1A* | *PRKDC* |
| *PTEN* | *PTPRD* | *PTPRT* | *RAD50* | *RAD51B* | *RAD51C* | *RAD51D* | *RAD54L* |
| *RAF1* | *RARA* | *RB1* | *RBM10* | *RET* | *RNF43* | *ROS1* | *RUNX1* |
| *SETD2* | *SMAD4* | *SMARCA4* | *SOX2* | *SOX9* | *SPOP* | *SPTA1* | *SRC* |
| *STAG2* | *STK11* | *TBX3* | *TERT* | *TGFBR2* | *TP53* | *TP63* | *TRIM58* |
| *TRPC5* | *U2AF1* | *UGT1A1* | *VEGFA* | *VEGFB* | *VEGFC* | *VHL* | *YES1* |
